# Draft Genome Sequence of *Saccharibacillus* sp. Strain WB 17, Isolated from Wheat Phyllosphere

**DOI:** 10.1128/MRA.01201-19

**Published:** 2020-02-13

**Authors:** Ludovic Besaury, Caroline Remond

**Affiliations:** a FARE Laboratory, Chaire AFERE, Université de Reims Champagne-Ardenne, Chaire AFERE, INRA, UMR A 614 FARE, Reims, France; University of Maryland School of Medicine

## Abstract

The whole genome of *Saccharibacillus* sp. strain WB 17, a bacterial strain isolated from wheat phyllosphere, has been sequenced. This microorganism is equipped with several carbohydrate-active enzymes, which would explain its ability to fractionate lignocellulose.

## ANNOUNCEMENT

The phyllosphere represents a unique niche in which microorganisms have acquired the ability to degrade lignocellulose (1) in order to survive oligotrophic conditions. Among microorganisms recovered from the phyllosphere, bacteria affiliated with the *Paenibacillaceae* family and the *Saccharibacillus* genus are present (2). *Saccharibacillus* sp. strain WB 17 was recovered from the culture of a wheat bran phyllosphere sampled in January 2018 from the Champagne-Ardennes area of France. The culture was performed at 30°C on 1× M3 medium (3) supplemented with wheat bran, under aerobic conditions. *Saccharibacillus* sp. WB 17 was identified based on its 16S rRNA gene sequence and was associated with the *Saccharibacillus* genus. To further characterize the metabolic potential of *Saccharibacillus* sp. WB 17 and its ability to fractionate lignocellulose, its whole genome was sequenced. *Saccharibacillus* sp. WB 17 was grown for 48 h at 30°C on Luria-Bertani medium, and its genomic DNA was extracted using the PureLink genomic DNA minikit (Thermo Fisher Scientific). Whole-genome shotgun sequencing (2 × 150 bp) was performed using the Nextera DNA sample preparation kit (Illumina, San Diego, CA, USA) following the manufacturer’s user guide and sequenced on a NovaSeq system (MR DNA [Molecular Research], Shallowater, TX, USA). In total, 30,007,734 reads were obtained. The sequence data files were filtered for quality using FastQC (4) and then *de novo* assembled by SOAPdenovo (version 2.04) (5); default parameters were used for all software. A total of 47 contigs were detected, with sequencing coverage of 409-fold. The *N*_50_ value was 205,341 bp. The size of the assembled genome was 5,391,836 bp. The genome size of the strain was between the sizes of the two closest *Saccharibacillus* relatives (6.08 Mbp for Saccharibacillus sacchari GR21^T^ and 4.69 Mbp for Saccharibacillus kuerlensis HR1^T^). *Saccharibacillus* sp. WB 17 had a G+C content of 58.82%. This value was in the range of known values for *Saccharibacillus* genomes. Indeed, the G+C content values recorded for the previously sequenced genomes were as follows: 58.4 mol% (Saccharibacillus qingshengii H6^T^) (6), 57.8 mol% (S. sacchari GR21^T^) (7), 50.5 mol% (S. kuerlensis HR1^T^) (8), and 55.5 mol% (Saccharibacillus deserti WLJ055^T^) (9). The draft genome of *Saccharibacillus* sp. WB 17 was annotated by the NCBI Prokaryotic Genome Annotation Pipeline (PGAP) (https://www.ncbi.nlm.nih.gov/genome/annotation_prok); it contained 73 tRNAs, 4,826 genes, and 4,730 coding sequences (CDSs). Only 1,139 CDSs were annotated, which represented 22% of the genome content. Based on the Carbohydrate-Active EnZymes database (CAZy) database (10), the genome coded for a total of 236 carbohydrate-active enzymes in five categories, namely, glycoside hydrolases (145 CDSs), glycosyl transferases (31 CDSs), polysaccharide lyases (3 CDSs), carbohydrate esterases (31 CDSs), and carbohydrate-binding modules (21 CDSs); however, no auxiliary activities (redox enzymes that act in conjunction with carbohydrate-active enzymes) were detected.

### Data availability.

The genome data for *Saccharibacillus* sp. strain WB 17 are available in GenBank under accession number VPFK00000000 and SRA accession number SRR10389757. The version reported in this paper is the second version, VPFK00000000.2. The 16S rRNA data are available under accession number MN475752.

## References

[1] Voříšková J, Baldrian P 2013 Fungal community on decomposing leaf litter undergoes rapid successional changes. ISME J 7:477–486. doi:10.1038/ismej.2012.116. 23051693 PMC3578564

[2] Mayilraj S, Stackebrandt E 2014 The family Paenibacillaceae, p 267–280. *In* Rosenberg E, DeLong EF, Lory S, Stackebrandt E, Thompson F (ed), The prokaryotes: Firmicutes and Tenericutes, 4th ed Springer, Berlin, Germany.

[3] Vieira F, Nahas E 2005 Comparison of microbial numbers in soils by using various culture media and temperatures. Microbiol Res 160:197–202. doi:10.1016/j.micres.2005.01.004. 15881837

[4] Andrews S 2010 FastQC: a quality control tool for high throughput sequence data. Babraham Bioinformatics, Babraham Institute, Cambridge, United Kingdom.

[5] Luo R, Liu B, Xie Y, Li Z, Huang W, Yuan J, He G, Chen Y, Pan Q, Liu Y, Tang J, Wu G, Zhang H, Shi Y, Liu Y, Yu C, Wang B, Lu Y, Han C, Cheung DW, Yiu S-M, Peng S, Xiaoqian Z, Liu G, Liao X, Li Y, Yang H, Wang J, Lam T-W, Wang J 2012 SOAPdenovo2: an empirically improved memory-efficient short-read *de novo* assembler. GigaScience 1:18. doi:10.1186/2047-217X-1-18. 23587118 PMC3626529

[6] Han H, Gao S, Wang Q, He L-Y, Sheng X-F 2016 *Saccharibacillus qingshengii* sp. nov., isolated from a lead-cadmium tailing. Int J Syst Evol Microbiol 66:4645–4649. doi:10.1099/ijsem.0.001404. 27514529

[7] Rivas R, García-Fraile P, Zurdo-Piñeiro JL, Mateos PF, Martínez-Molina E, Bedmar EJ, Sánchez-Raya J, Velázquez E 2008 *Saccharibacillus sacchari* gen. nov., sp. nov., isolated from sugar cane. Int J Syst Evol Microbiol 58:1850–1854. doi:10.1099/ijs.0.65499-0. 18676467

[8] Yang S-Y, Liu H, Liu R, Zhang K-Y, Lai R 2009 *Saccharibacillus kuerlensis* sp. nov., isolated from a desert soil. Int J Syst Evol Microbiol 59:953–957. doi:10.1099/ijs.0.005199-0. 19406774

[9] Sun J-Q, Wang X-Y, Wang L-J, Xu L, Liu M, Wu X-L 2016 *Saccharibacillus deserti* sp. nov., isolated from desert soil. Int J Syst Evol Microbiol 66:623–627. doi:10.1099/ijsem.0.000766. 26559492

[10] Cantarel BL, Coutinho PM, Rancurel C, Bernard T, Lombard V, Henrissat B 2009 The Carbohydrate-Active EnZymes database (CAZy): an expert resource for glycogenomics. Nucleic Acids Res 37:D233–D238. doi:10.1093/nar/gkn663. 18838391 PMC2686590

